# Molecular mechanisms of tumor resistance to radiotherapy

**DOI:** 10.1186/s12943-023-01801-2

**Published:** 2023-06-15

**Authors:** Yu Wu, Yingqiu Song, Runze Wang, Tianlu Wang

**Affiliations:** 1 Department of Radiotherapy, Cancer Hospital of China Medical University, Liaoning Cancer Hospital & Institute, Cancer Hospital of Dalian University of Technology, No.44 Xiaoheyan Road, Dadong District, Shenyang, 110042 Liaoning Province China; 2 School of Graduate, Dalian Medical University, Dalian, 116044 China; 3 Faculty of Medicine, Dalian University of Technology, Dalian, 116024 China

**Keywords:** Cancer, Radiotherapy, Tumor resistance

## Abstract

**Background:**

Cancer is the most prevalent cause of death globally, and radiotherapy is considered the standard of care for most solid tumors, including lung, breast, esophageal, and colorectal cancers and glioblastoma. Resistance to radiation can lead to local treatment failure and even cancer recurrence.

**Main body:**

In this review, we have extensively discussed several crucial aspects that cause resistance of cancer to radiation therapy, including radiation-induced DNA damage repair, cell cycle arrest, apoptosis escape, abundance of cancer stem cells, modification of cancer cells and their microenvironment, presence of exosomal and non-coding RNA, metabolic reprogramming, and ferroptosis. We aim to focus on the molecular mechanisms of cancer radiotherapy resistance in relation to these aspects and to discuss possible targets to improve treatment outcomes.

**Conclusions:**

Studying the molecular mechanisms responsible for radiotherapy resistance and its interactions with the tumor environment will help improve cancer responses to radiotherapy. Our review provides a foundation to identify and overcome the obstacles to effective radiotherapy.

## Introduction

Cancer incidence and mortality are increasing globally, making it one of the greatest challenges threatening human health and decreasing life expectancy. According to the most recent World Health Organization/International Agency for Research on Cancer (WHO/IARC) Global Cancer Report, there will be approximately 19.3 million new cancer cases and approximately 10 million deaths worldwide in 2020. Additionally, it is predicted that there may be a 60% increase in cancer cases over the next 20 years [1]. Clinical cancer rates are increasing in almost all countries, and it is anticipated that in 2023, there will be approximately 1,958,310 new cases of cancer in the United States, which is equivalent to more than 5,000 new cases per day. Although mortality from cancer has decreased slightly with advances in early prevention, screening, diagnosis, and treatment methods [2], comprehensive global cancer data show that increased effort is required to further decrease cancer mortality. Surgery, radiation, chemotherapy, immunotherapy, targeted therapy, stem cell transplantation, and multidisciplinary team therapy are all used to treat cancer [3]. Radiation therapy (RT), one of the three traditional components of cancer treatment, is particularly effective at removing or controlling certain tumors when it is combined with surgery, chemotherapy, immunotherapy, and other treatments [4, 5]. Theoretically, radiotherapy should be effective for all tumor cells; however, each cell has a different sensitivity to radiation, leading to different treatment effects [6]. Radiation resistance, which results in RT failure, metastasis, cancer recurrence, and a poor prognosis, continues to be a major obstacle in improving treatment outcomes despite the development of novel radiotherapy techniques and the adoption of new treatment approaches [5, 7]. Radiotherapy resistance can occur in several ways, as it is related to the heterogeneity of the tumor and the surrounding microenvironment as well as numerous gene alterations [8–11]. Thus, it is essential to assess the mechanisms behind the development of resistance in solid tumors to both conventional and new RTs.

In this review, we aim to discuss the mechanisms currently associated with radioresistance and to summarize potential targets that may be used to increase cancer radiosensitivity (Table 1).

**Table 1:** Mechanisms of cancer cell resistance to radiotherapy

Mechanisms	Mediators	References
DNA damage repair	ATM DNA-PKcs PARPs rH2AX WEE1 BRCA1 MDC1 53BP1 NBS1/hMRE11/hRAD50 Ku70/Ku80	[12–21]
Cell cycle redistribution	ATM ATR Chk1 Chk2 P53 P21	[22–31]
Apoptosis escape	Bcl family Pro-survival pathways IAPs	[32–34]
Microenvironment	Hypoxia	[33]
	Inflammation	[35]
	Immunosuppression	[36–50]
	Cancer-associated fibroblast	[51–63]
CSCs	High survival (autophagy, apoptosis, DNA damage repair)	[64, 65]
	Generates immunosuppressive signal	[44]
	Low ROS levels	[66, 67]
	Epithelial–mesenchymal transition (high plasticity)	[68]
	Quiescence	[69]
	Signaling pathway (Wnt Notch Hedgehog TGF-ß PI3K/AKT/mTOR)	[37–39, 70, 71]
Metabolic reprogramming	Glucose metabolism	[72, 73]
	Lipid metabolism	[74–76]
	High expression of glutamine synthetase, purine, and serine protease inhibitor E2	[77–79]
Exosomes	M1-M2 TAM polarization	[80]
	ncRNA	[81–86]
Ferroptosis	GPX4 PUFA-PL CoQ-FSP1 Iron metabolism	[87–90]

*ATM *ataxia-telangiectasia mutated, *DNA-PKcs *DNA-dependent protein kinase catalytic subunit, *CHK1 *checkpoint kinase 1, *CHK2 *checkpoint kinase 2, *PARP *poly(ADP-ribose) polymerase, *ATR *ATM and Rad3-related kinase, *DNA-PKcs *DNA-dependent protein kinase, *IAPs *Inhibitor of apoptosis proteins, *M1 *M1-type tumor-associated macrophages (anti-tumor), *M2 *M2-type tumor-associated macrophages (pro-tumor), *GPX4 *glutathione peroxidase 4, *PUFA-PL *polyunsaturated fatty acid- containing phospholipid, *ROS *reactive oxygen species, *BRCA1 *Breast Cancer Susceptibility Protein-1, *MDC1* mediator of DNA damage check point protein 1, *BRCA1* Breast Cancer Susceptibility Protein-1

## Molecular mechanisms of cancer radioresistance

### DNA damage repair

**Fig. 1:**
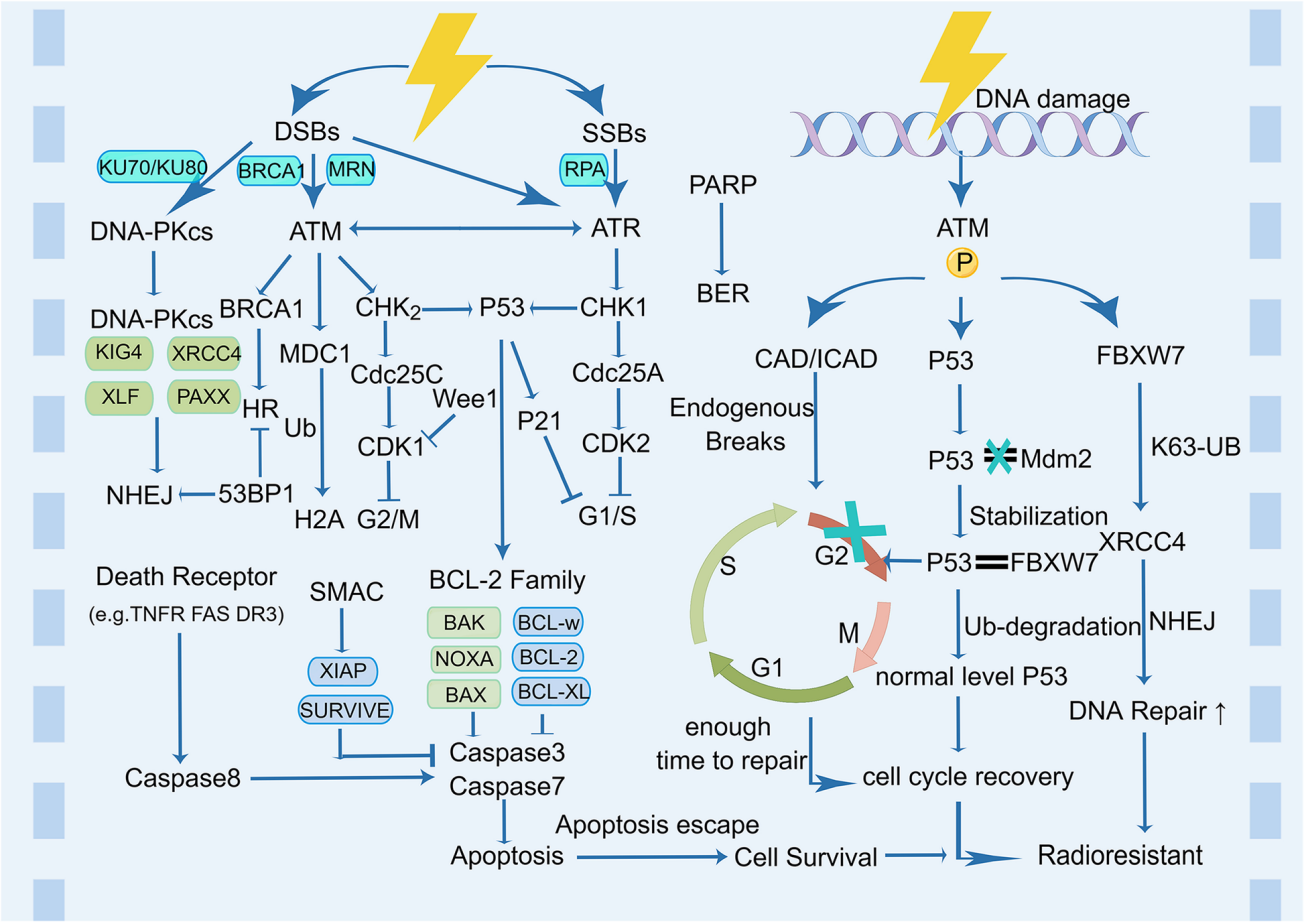
The multiple pathways for DNA damage repair, cell cycle arrest, and apoptosis escape after radiation therapy. Key regulators in the DNA damage repair pathway may alter sensitivity to radiotherapy in cancer cells, whereas cell cycle checkpoints may respond to damage when tumor cells are exposed to ionizing radiation, thus causing cell cycle arrest and allowing more time for repair, which increases resistance to radiotherapy. If DNA damage repair is unsuccessful, apoptotic signaling pathways are induced to resist radiotherapy damage. HR: homologous recombination, NHEJ: non-homologous end joining, BER: base excision repair DSBs: double-strand breaks, SSBs: single-strand breaks, ATM: ataxia-telangiectasia mutated, ATR: ATM and Rad3-related kinase, DNA-PKcs: DNA-dependent protein kinase, MRN: Mre11–Rad50–NBS1, RPA: replication protein A, DNA-PKcs: DNA-dependent protein kinase catalytic subunit, CHK1: checkpoint kinase 1, CHK2: checkpoint kinase 2, PARP: poly(ADP-ribose) polymerase, XRCC4: X-ray repair cross-complementing protein 4, XLF:XRCC4-like factor, PAXX: Paralogue of XRCC4 and XLF, LIG4: DNA ligase IV, MDC1: mediator of DNA damage checkpoint protein 1, CAD: caspase-activated DNase, ICAD: inhibitor of CAD, MDM2: mouse double minute 2 homolog, FBXW7: F-box and WD repeat domain-containing 7, BCL-2: B-cell lymphoma 2

Genotoxic cancer therapy inactivates and kills cancer cells via extensive DNA damage, and RT is the most widely used genotoxic challenge in standard oncology treatments. Ionizing radiation exerts both direct and indirect effects on DNA damage [91]. The direct effect is that DNA is damaged by directly absorbing the radiation energy, whereas the indirect effect is that other molecules around the DNA absorb the radiation energy and produce abnormally active free radicals that interact with DNA and other large molecules to cause damage [92]. When radiation passes through genetic material, the deposition of energy triggers extensive DNA damage; usually, this type of damage is in the form of double-strand breaks (DSBs), single-strand breaks, base damage, and interstrand crosslinks, of which the most deleterious are DSBs [93]. These injuries may pose an insurmountable barrier to the adaptation of cancer cells and promote tumor cell demise [91]. However, a complex and precise set of regulatory mechanisms have evolved to deal with these types of damage, primarily numerous repair pathways, such as mismatch repair, base excision repair, nucleotide excision repair, and DSB repair. Non-homologous end joining (NHEJ) and homologous recombination are two key modalities of DSB repair. DNA damage checkpoints are activated simultaneously, which delay the onset of mitosis and provide more time for DNA repair [94–96]. During the evolution of cancer cells, multiple integrated molecular signals lead to increased tumor cell resistance to radiotherapy, resulting in radiotherapy failure. Therefore, understanding how cells activate and implement DNA damage repair pathways is crucial to preventing tumor cell DNA repair and, thus, the induction of tumor cell necrosis and apoptosis. DNA damage sensors such as ATRIP, Rad24p, γH2AX, NBS1, BRCA1/2, Ku70/80, and RNA polymerase recognize damage signals, recruit the DNA damage response (DDR) core kinase “ataxia-telangiectasia mutated” (ATM), “ATM- and Rad3-Related” (ATR), DNA-dependent protein kinase (DNA-PK), and other regulatory factors to DNA break sites, and catalyze the activation of a variety of downstream signaling molecules, thus promoting DNA damage repair [12–14, 97, 98]. Researchers have discovered that overexpression of the Mre11-Rad50-Nbs1 complex in rectal cancer markedly increases radioresistance and is associated with a poor prognosis. The Mre11-Rad50-Nbs1 (MRN) complex plays a crucial role in recognizing and initiating the DSB repair pathway [15]. H2AX can detect the genotoxic effects of various toxic substances, monitor the clinical side effects of radiotherapy, and predict changes in the sensitivity of cancer cells to RT. The number of histone -H2AX (-H2AX) foci correlates with the number of radiation-induced DSBs [16, 17]. Increased Ku70/80 expression has been substantially associated with radioresistance, and a considerable increase in Ku expression occurs in advanced rectal cancer [18, 19]. ATM recruits phosphorylated FBXW7 to DSB sites, whereas activated DNA-PKcs phosphorylate XRCC4. The SCF(FBXW7) E3 ligase activates XRCC4 via ubiquitination to facilitate binding to the Ku70/80 complex, thereby enhancing NHEJ repair and leading to radiotherapy tolerance [20]. Therefore, these sensor and effector response cascades in response to DNA damage may serve as valuable and sensitive markers that can predict the clinical radiotherapy outcomes for certain cancers. The susceptibility of cancer cells to RT and the selected repair process change with changes in the genes and proteins involved in DNA damage and repair, translocations, interactions, and mutual regulation. An important research technique to increase the efficacy of tumor therapy is to target key regulators in the DDR pathway of tumor cells and to reduce the tolerance of tumor cells to radiotherapy by disrupting the DDR regulatory system (Fig. 1).

#### DNA-PKs

Tumor cells engage in classical NHEJ for repair primarily via DNA-PKs [21]. DSBs caused by radiation damage are recognized and recruit enzymes involved in the DNA damage repair process, such as PNKP, Tdp-1, and APE-1, and these proteins recruit the XPCC4-XLF-LIG4 complex, which eventually joins the broken DNA ends together [99, 100]. DNA-PKcs are primarily localized in the nucleus and phosphorylate themselves and a range of downstream targets, including XRCC4 [101]. Cells lacking DNA-PKc exhibit higher radiosensitivity. In patients with multiple myeloma, the upregulation of DNA-PKcs can be observed in association with radioresistance [102]. According to a glioblastoma (GBM) study, DNA-PK stabilizes SOX2 by phosphorylating it, which promotes treatment resistance by malignantly progressing glioma stem-like cells (GSCs) in a stem cell state. Inhibiting DNA-PK in mice also causes glioma stem cell differentiation and sensitizes the GBM to radiation [103]. Wang et al. demonstrated using both in vitro and in vivo experiments that LINC-PINT interacted with DNA-PKcs, inhibited the recruitment of DNA-PKcs at DNA damage sites, decreased the level of DNA damage repair factors, and increased the radiosensitivity of nasopharyngeal carcinoma [104]. This team also discovered and validated that nasopharyngeal carcinoma cells specifically bind DNA-PKcs via linc00312 and promote its degradation, reducing DNA damage signaling and exhibiting radiosensitizing effects; they also found that patients with low levels of DNA-PKc expression had significantly increased survival at their follow-up examinations [105]. This information suggests that the increased activity of DNA-PKcs can, to a certain extent, inhibit cell death and increase tolerance to radiotherapy. Inhibition of DNA-PKcs has been shown to exacerbate the cytotoxic effects of radiotherapy, and the oral DNA-PKcs inhibitor M3814 induced considerable sensitization to radiotherapy in preclinical models [106]. Moreover, researchers demonstrated through a series of ex vivo and in vivo experiments that the DNA-PKcs inhibitor AZD7648 alone or in combination with radiotherapy improves the anti-tumor activity and treatment response in a variety of cancer cell types and xenograft models, respectively [107]. In addition, inhibition of other pathway nodes affecting the activation of DNA-PKcs DSB repair, including but not limited to ERK and MEK, significantly enhanced the radiosensitivity of tumor cells [108–111]. Thus, DNA-PKcs and multiple loci affecting its activity are considered promising targets for overcoming radiotherapy resistance interventions.

#### PARP

Nuclease PARP participates actively in numerous DNA repair pathways and helps preserve genomic integrity [112, 113]; however, it is known for its crucial function in the restoration of DNA single-strand breaks. The ZnF structural domain of PARP rapidly recognizes and binds to the break site when single-strand breaks occur in radiation-damaged DNA and recruits repair proteins such as XRCC1 to the damage site, thereby repairing single-strand breaks, which increases the resistance of cancer cells to radiation [114]. Inhibiting PARP and its base excision repair leads to an accumulation of unrepaired single-strand breaks, and its eventual collision with a traveling DNA replication fork will translate into DNA DSBs [115]. Researchers found that BRCA1/2 mutant cells were significantly more sensitive to PARP inhibitors than the mutant phenotype, and then went on to confirm a "synthetic lethal" effect between homologous recombination repair defects and PARP inhibitors [116, 117]. PARP inhibitors can increase the biological impact of RT by impeding the ability of cancer cells to repair DNA damage. To effectively radiosensitize a variety of cancer types, including breast, prostate, and pancreatic tumors with BRCA1/2 mutations, clinicians design a treatment plan that combines ionizing radiation and PARP inhibitors [118–120]. A clinical trial in triple-negative breast cancer showed that radiotherapy combined with PARP inhibitors in the setting of BRCA1/2 mutations improved the efficacy of radiotherapy by indirectly increasing the frequency of unrepaired DSBs via the base excision repair pathway, and it showed good safety and tolerability [121]. The PARP inhibitors olaparib and niraparib were found to block DNA damage repair in the cancer cells of ovarian cancer patients and made cancer cells more sensitive to radiotherapy [122, 123]. PARP-1-targeted radiotherapy in a murine model of GBM was effective, and the tumor model mice in the 131I-PARPi treatment group had a longer survival time than the control mice [124]. The PARP-1/-2 inhibitor MK-4827 sensitizes human breast cancer xenografts to radiotherapy in cellular assays [125]. Further studies revealed that nitric oxide (NO) donors could block BRCA expression and thus inhibit HRR, and that PARP inhibitors could provide a new targeting option for radiosensitization in patients with normal BRCA1/2 genes by cascading with NO donors [126]. These findings imply that PARPs play a crucial role in tumor RT tolerance and hold promise for successful applications in a wider range of cancer therapies that target PARPs, allowing the development of novel combination therapies to overcome cancer drug resistance.

### Cell cycle arrest

There are four distinct phases of the cell cycle: G1 (a growth phase preceding DNA synthesis), S (DNA replication/synthesis), G2 (final preparation for division phase), and M (mitosis) [127, 128]. The G1/S phase and G2/M phase checkpoints are crucial for the cell cycle and can respond to disruptions or damage by blocking the cell cycle [22]. When ionizing radiation damages tumor cells, they block the cell cycle to buy valuable time for self-repair and to escape from radiotherapy damage, thus increasing resistance to radiotherapy [23]. Because of the presence of the G1/S checkpoint key regulator p53, normal cells will stagnate in the G1 phase (pre-DNA synthesis) after DNA damage and not enter S phase (DNA synthesis phase) and initiate DNA damage repair mechanisms. However, in tumor cells, G1/S checkpoint regulators are often out of action, allowing tumor cells to easily enter the S phase [24]. Recent studies have reported that cancer cells, when exposed to radiation damage, cause their own DNA breaks by activating Caspase-activated Dnase (CAD) expression, which promotes G2 phase arrest during interphase cell division, buying time for DNA damage caused by RT. Further studies found that inhibition of CAD contributes to radiotherapy sensitivity [25]. Targeting the G2 cycle checkpoint pathway may be a potential way to improve the efficacy of radiotherapy.

#### ATM

ATM is a serine-threonine protein kinase that plays an important role in the ionizing radiation (IR)-induced DSB repair process, cell cycle checkpoint maintenance, and DNA damage repair [26]. After DNA damage occurs, the MRN complex, ATR, and Wip1 interact with ATM, and activated ATM is then recruited to the damaged DSB site to initiate cell cycle arrest by phosphorylating multiple substrates, thus providing time for DNA damage repair [27, 28]. If DNA damage cannot be repaired, it will trigger an apoptotic response [29–31]. Activation of ATM catalyzes the activation of multiple downstream targets such as CHK2, NBS1, and BRCA1, of which CHK2 is one of the most important substrates that can regulate the phosphorylation of molecules such as CDC25c and P53, finally mediating cell cycle arrest. ATM regulates p53 levels and function by phosphorylating CHK2, which in turn inhibits CDK2 via p21 expression and induces G1 phase arrest. Furthermore, CHK2 phosphorylated by ATM can also phosphorylate its key substrate, protein phosphatase CDC25c, and inhibit its function to induce G2 phase arrest. Activated ATM is involved in S phase checkpoint regulation via phosphorylation of BRCA1 or NBS1, which mediates S phase block [31, 129–131]. Because of its involvement in boosting DNA damage repair and causing cell cycle arrest, studies have demonstrated that high levels of ATM expression may be connected with resistance to radiation [30]. The inhibition of ATM expression, which prevents ATM from inducing the activation of ATM-CHK2 and ATR-CHK1 signaling cascades, improves the sensitivity of tumor cells to RT [132, 133]. The mechanism of action related to the ATM regulation of P53, which corresponds to apoptosis after radiotherapy and has a crucial role in radiation-induced cellular effects, has also received a large amount of attention [134, 135]. In response to the damage caused by ionizing radiation, activated ATM phosphorylates P53, separates the binding of P53 to the negative regulator MDM2, causes cell cycle arrest, and promotes the binding of FBXW7 to P53, thus mediating the degradation of p53 to ensure that the accumulated p53 is restored to basal levels; this allows cancer cells to resume normal cell cycle progression and confers radioresistance [136]. In summary, ATM, as an important node in cell cycle arrest and apoptosis, may reduce radiation-induced resistance to radiotherapy by suppressing the ATM gene, and is thus a successful tactic by which to enhance the therapeutic impacts of cancer treatments and enhance patient prognosis.

### Apoptosis

Apoptosis is a type of controlled cell death that is independent, organized, and dictated by multiple genes. It can occur through an endogenous pathway involving Bcl-2-mediated mitochondrial cytochrome C or an exogenous pathway mediated by death receptor ligand expression [137]. "Apoptosis evasion," defined as the third feature of cancer cells, is an important survival ability of these cells for protection against radiation damage when DNA damage repair is unsuccessful [138]. Tumor cells have developed several mechanisms to prevent apoptosis and resist RT and survive. Radiation-resistant cancer cells normally inhibit apoptosis by regulating the Bcl family interaction network [32, 35]. The upregulation of anti-apoptotic proteins, such as Bcl-2 and Bcl-XL, and the downregulation or inactivation of pro-apoptotic proteins, such as Bax and Bak, are some strategies to avoid apoptosis and increased radiation resistance [139–142]. Targeting important regulators of the Bcl-2 protein family has been shown to overcome cancer resistance to apoptosis in multiple cancer types as a method of radiosensitization [36]. Recent studies have shown that combining Bax activators and Bcl-XL inhibitors significantly enhances apoptosis and disrupts tumor therapeutic resistance [143]. Ma et al. investigated the effect of RBM3 on nasopharyngeal carcinoma cell radioresistance and found that it activates the survival PI3K/AKT signaling pathway, regulates Bcl-2, and inhibits caspase 3, enabling cancer cell survival through apoptosis evasion and increasing resistance to RT [33]. Other pro-survival pathways, such as RAS/MEK/ERK, have also been found to be activated in cancer cells to control apoptosis for similar purposes [144–147]. Therefore, these pathways have emerged as attractive targets for the development of cancer therapies. Sun and his team discovered that the mimetic E2-coupled enzyme UBE2F activates CRL5 degrades the substrate pro-apoptotic protein NOXA, and inhibits tumor cell apoptosis [148]. Subsequently, his team independently developed the small-molecule inhibitor HA-9104 targeting the UBE2F-CRL5 axis, which promoted a large accumulation of NOXA in lung cancer cells, inducing apoptosis and enhancing their sensitivity to RT [149]. The inhibitor of apoptosis proteins (IAPs) are key regulators of apoptosis, and to date, eight members of this protein family have been identified: neuronal IAP (NIAP), cellular IAP1 (c-IAP1), cellular IAP2 (c-IAP2), X-chromosome-linked IAP (XIAP), survivin, ubiquitin-binding BIR structural domain enzyme (BRUCE), melanoma IAP (ML-IAP), and IAP-like protein 2 (ILP2). According to a study, overexpressing apoptosis inhibitory proteins may result in faulty apoptosis and increase cancer cell resistance to radiation treatment [150]. For instance, in rectal cancer cells, increased survivin levels reduced apoptosis and improved radioresistance [151]. X-linked IAP (endogenous mitochondrial pathway) and cIAP1/2 (exogenous TNF receptor pathway) are overexpressed in head and neck squamous cell carcinoma (HNSCC) cells, increasing apoptosis resistance and reducing the sensitivity of cancer cells to radiotherapy by blocking downstream caspase activity that is essential for the internal and external apoptotic pathways [34]. A new double-blind, randomized phase 2 clinical study by Jean and colleagues showed that Xevinapant (a novel potent small-molecule IAP antagonist that inhibits XIAP and cIAP1/2) plus RT reduced the risk of death by more than half compared to placebo plus RT in patients with unresectable, locally advanced HNSCC, while not increasing toxicity. This supports that standard RT combined with targeting an important regulator of apoptosis may improve cancer therapy outcomes [152]. Pivotal phase 3 studies of treatment with Xevinapant plus chemoradiotherapy for patients with unresected LA SCCHN (Trilynx) and Xevinapant plus RT for patients with resected LA SCCHN (XRay Vision) are currently underway to explore greater possibilities [153]. Hence, exploring biomarkers of tumor apoptosis evasion and using combination therapy targeting multiple features may be an effective strategy to achieve radiosensitization.

### Tumor microenvironment

**Fig. 2:**
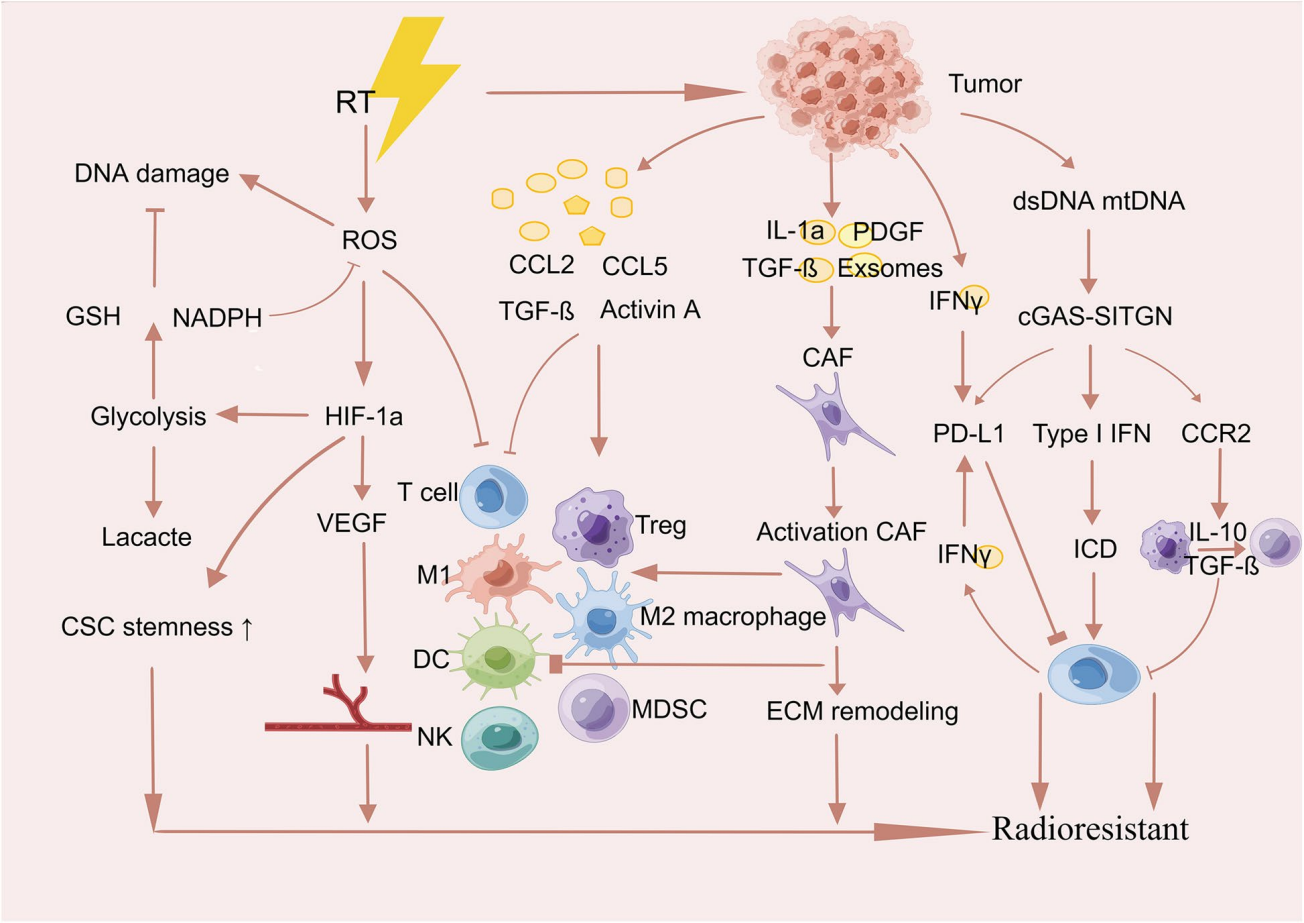
The development of radioresistance in the tumor microenvironment after radiation. Radiation can induce hypoxia, fibrosis, vascular damage, chronic inflammation, and immunosuppression in the tumor microenvironment, all of which may lead to RT resistance. Cancer-associated fibroblasts are also important aspects of the tumor microenvironment for the generation of radioresistance because they affect immune cells in such a way that leads to immunosuppression, fibrosis, and extracellular matrix remodeling. RT: radiation therapy, ROS: reactive oxygen species, GSH: glutathione, VEGF: vascular endothelial growth factor, Treg: regulatory T cells, NK: natural killer cells, DC: dendritic cells, CAF: tumor-associated fibroblasts, MDSCs: bone-marrow-derived suppressor cells, ECM: extracellular matrix, IL-1α: interleukin-1α, HIF-1α: hypoxia-inducible factor 1α, PDGF: Platelet-derived growth factor, CSC: Cancer stem cell, NADPH: nicotinamide adenine dinucleotide phosphate oxidase, TGF-β: transforming growth factor-β, CHK1: checkpoint kinase 1, CHK2: checkpoint kinase 2, cGAS: cyclic GMP-AMP synthase, STING: stimulator of interferon genes, IFN: interferon, PD‑L1: programmed cell death 1 ligand 1, IL‑10: interleukin 10, ICD: immunogenic cell death

The tumor microenvironment, which is the area in which tumors are present, consists of tumor cells, extracellular matrix, chemokines, cytokines, and other molecules and is characterized by hypoxia and a low pH [51, 52]. Changes in the tumor microenvironment are inextricably linked to the growth, invasion, and spread of tumor cells and resistance to treatment [53]. Radiation can induce chronic inflammation, fibrosis, hypoxia, vascular damage, and immunosuppression in the tumor microenvironment and enhance the pro-inflammatory response [54, 55]. Cancer cells release pro-inflammatory factors such as IL-6, IL-1a, TGF-β, and TNF-ɑ to activate cancer-associated fibroblasts (CAF) into iCAF [56], and radiation induces tumor cells to secrete large amounts of cytokines with radioresistance [57, 58]. Radiation also increases tumor hypoxia, reduces oxygen-dependent DNA damage, and induces HIF-1–mediated cell survival [59, 60]. Furthermore, it also induces an increase in reactive oxygen species (ROS) levels, which mediate HIF-1 stabilization and promote angiogenesis. Radiation can thus aid tumor survival by creating hypoxic conditions that limit tumor-eliminating effector immune cells and promote the activation of immunosuppressive cells, thus creating an immunosuppressive microenvironment that contributes to radioresistance [61]. A better understanding of the unique microenvironmental interactions of tumors will help to improve the efficacy of radiotherapy (Fig. 2).

#### Hypoxia effect

Hypoxia is a crucial tumor growth regulator and is essential for RT resistance [62]. The resistance of tumor cells to radiation can be increased 2–3 times in a hypoxic environment [63]. Studies have shown that hypoxic conditions activate signaling pathways involved in the epithelial–mesenchymal transition (EMT) process, thereby promoting EMT and increasing tumor resistance [154]. Hypoxia can also induce hypoxia-inducible factors, promote VEGF secretion by tumor cells, promote tumor vascular regeneration, protect vascular endothelial cells, and antagonize the cytotoxic effects of radiation [155]. Activated HIF-1 acts on key glycolytic enzymes to produce NADPH and glutathione to scavenge ROS generated after radiation, thereby reducing DNA damage while secreting large amounts of lactate [156, 157]. Yang et al. found that lactate molecules could boost tumor radiation resistance by promoting the functional activation of myeloid-derived suppressor immune cells (MDSCs) in the microenvironment via the GPR81/mTOR/HIF-1/STAT3 pathway [158]. One study suggested that hypoxia can make osteosarcoma cells more resistant to radiation by triggering autophagy, which increases the removal rate of cellular ROS products,HIF-1 and HIF-2 expression levels were negatively correlated with the effects of radiotherapy on osteosarcoma [68]. Intra-tumor hypoxia can maintain stem cell traits in tumor stem cells, leading to radiotherapy resistance [159]. Furthermore, a recent study revealed that oxygen deprivation induced significant ANGPT4 protein expression in lung cancer cells, and the degree of its expression was positively linked with radiation resistance; furthermore, oxygen deprivation also promoted the expression of iron death inhibitory proteins such as GPX4, further increasing lung cancer radiation resistance [160]. In addition, early preclinical investigations have demonstrated the efficacy of hyperbaric oxygen, carbogen with nicotinamide, and nitroimidazole in boosting radiation sensitization during hypoxia restriction; however, clinical trials have been constrained due to local control rates, toxicity, and operability [64, 161–165]. Nevertheless, hypoxia-activated prodrugs such as nimorazole have shown significant radiosensitizing effects in clinical trials enrolling patients with head and neck cancer and supraglottic laryngeal and pharyngeal carcinoma [166]. Recent studies found that the delivery of the tumor oxidant manganese dioxide nanoparticles (MnO2-NPs) and the HIF-1 functional inhibitor acridine flavonoid (ACF) into tumor tissues using a nanomedicine platform enhanced the effects of radiation therapy and distal effects [167]. These evidences indicate the importance of exploring targeted hypoxia and its combined strategies to improve radiotherapy sensitivity.

#### Cancer-associated fibroblasts

Cancer-associated fibroblasts are one of the most important and conspicuous plastic cell types in the tumor microenvironment, and they can be activated by a variety of cancer-associated active mediators (such as TGF-β, PDGF, FG, FNF-B, etc.) that are secreted by cancerous or immune cells. In turn, these cells then secrete a variety of cytokines, growth factors, chemokines, and extracellular matrix remodeling molecules to encourage tumor development [168–170]. CAF was found to promote the EMT of tumor cells, enhancing the ability of tumor cells to invade and metastasize and thus affecting the sensitivity of tumors to radiotherapy [69, 171]. CAF can also activate the stemness of tumor cells and induce resistance to radiotherapy via paracrine exosomes [172, 173]. Zhang et al. found that CXCL1 secreted by CAF regulates the DDR in an ROS-dependent manner and mediates radiation resistance [174]. Further studies reported that CAF also promotes the expression of lncRNA DNM3OS to regulate the DDR in esophageal squamous cell carcinomas with significant radioresistance [175]. A recent study illustrates that, after radiotherapy, tumor cells release large amounts of IL-1a to differentiate cancer-associated fibroblasts into inflammatory tumor-associated fibroblasts and cause oxidative-damage-mediated cellular senescence, leading to reduced radiotherapy sensitivity and tumor growth [176]. In terms of metabolism, CAF produces energy primarily via aerobic glycolysis while secreting large amounts of lactic acid, which affects the toxic effects of immune cells and leads to immunosuppression [65, 177]. In addition, CAF can reduce the anti-tumor effects of radiotherapy by causing an immunosuppressive microenvironment with an increased abundance of immunosuppressive cells and the suppression of effector immune cells [178–180]. As CAF is one of the main promoters of the tumor microenvironment that results in radiation resistance, targeting fibroblasts in the tumor microenvironment may be a possible therapeutic strategy to boost tumor sensitivity to radiotherapy.

#### Immune landscape

The first line of defense between immune cells and cancer cells is the tumor immune microenvironment, which is a crucial component of the tumor microenvironment [181]. RT has been shown to increase the immune system's resistance to tumors through a multitude of mechanisms, including producing a abscopal effect [66, 67], activating the cGAS-STING pathway, upregulating type I interferon transcription to promote the innate immune system [70], and inducing immunogenic death of tumors to trigger an antitumor adaptive immune response [71]. However, RT does not guarantee therapeutic efficacy or an immunological response. The explanation for this phenomenon relies on the ability of RT to stimulate not only the production of an anti-tumor immune response but also to activate the mechanism of tumor cell resistance to the synergistic immunity to radiotherapy.Using a tumor mouse model, researchers found that RT-mediated activation of STING/type I interferon signaling recruited MDSCs and increased resistance to RT, overcoming tumor immunogenicity. They also demonstrated CCR2, a monocyte chemokine, to be a potential target of RT to increase MDSC recruitment, and further experiments showed that the ability of radiation to kill cancer cells in mice was significantly enhanced when RT was combined with STING-activating drugs and anti-CCR2 antibodies [37]. MA and Liu's team found that high expression of the tumor cell-intrinsic E3 ligase TRIM7 was associated with poor prognosis in nasopharyngeal carcinoma by inhibiting mitochondrial DNA release and affecting STING-STING/1 type interferon signaling, thereby impairing CD8 T cell-mediated antitumor immune responses and causing resistance to radiation therapy [38]. Furthermore, ionizing radiation can upregulate PD-L1 expression through multiple pathways, reducing the toxic killing effect of CD8 + CTL on tumors [39, 40]. Combining radiation with anti-PD-L1 therapy was found to reduce immune escape and enhance the anti-tumor effects of RT [41]. Moreover, radiotherapy plus anti-CTLA-4 and other immunomodulatory treatments can work in concert [42, 43]. In addition, RT promotes the release of the immunosuppressive chemokines CCL2 and CCL5, activation of the immunosuppressive cytokine TGF-β, secretion of activator A, and local accumulation of extracellular adenosine, which together lead to the recruitment of regulatory T cells, immunosuppressive (M2) macrophages, and MDSCs, prevent CD8^+^ T cell activation and function, mediate tumor immune resistance, and inhibit RT effects [44–48]. Notably, TGF-β exhibits a dual function in the tumor microenvironment, inhibiting value-added induction of apoptosis at early stages and exerting a broad suppressive effect on the immune response through different mechanisms during tumor progression. The role of TGF-β in the tumor immune microenvironment and underlying mechanisms have been reviewed in detail elsewhere [49]. Inhibiting TGF-β can eliminate tumor microenvironment-mediated resistance to RT [50]. The use of Bintrafusp Alfa, a bifunctional fusion protein that inhibits both TGF-β and PD-L1, was found to synergize effectively with local radiotherapy (RT) in a variety of mouse immunocooled tumor models to overcome immune escape, eliminate treatment resistance, and improve survival while attenuating radiotherapy-induced fibrosis [182]. In addition, the RT-induced immune response may depend on the RT dose, as demonstrated in a study using a breast cancer mouse model, which showed that a single dose of radiation > 12–18 Gy induced the DNA exonuclease Trex1 to degrade dsDNA accumulated during RT to attenuate radiation-induced immunogenic cell death; in contrast, repeated irradiation < 12–18 Gy per dose increased immunostimulatory signals by activating the cGAS/STING pathway [72]. An in-depth comprehension of an investigation into the balance between the benefits and drawbacks of RT in the context of the tumor immune landscape may reveal potential targets for anti-cancer therapy, ideal radiation doses, and effective combinations of RT and immunotherapy to optimize treatment outcomes and boost RT effectiveness.

### Tumor stem cells

**Fig. 3:**
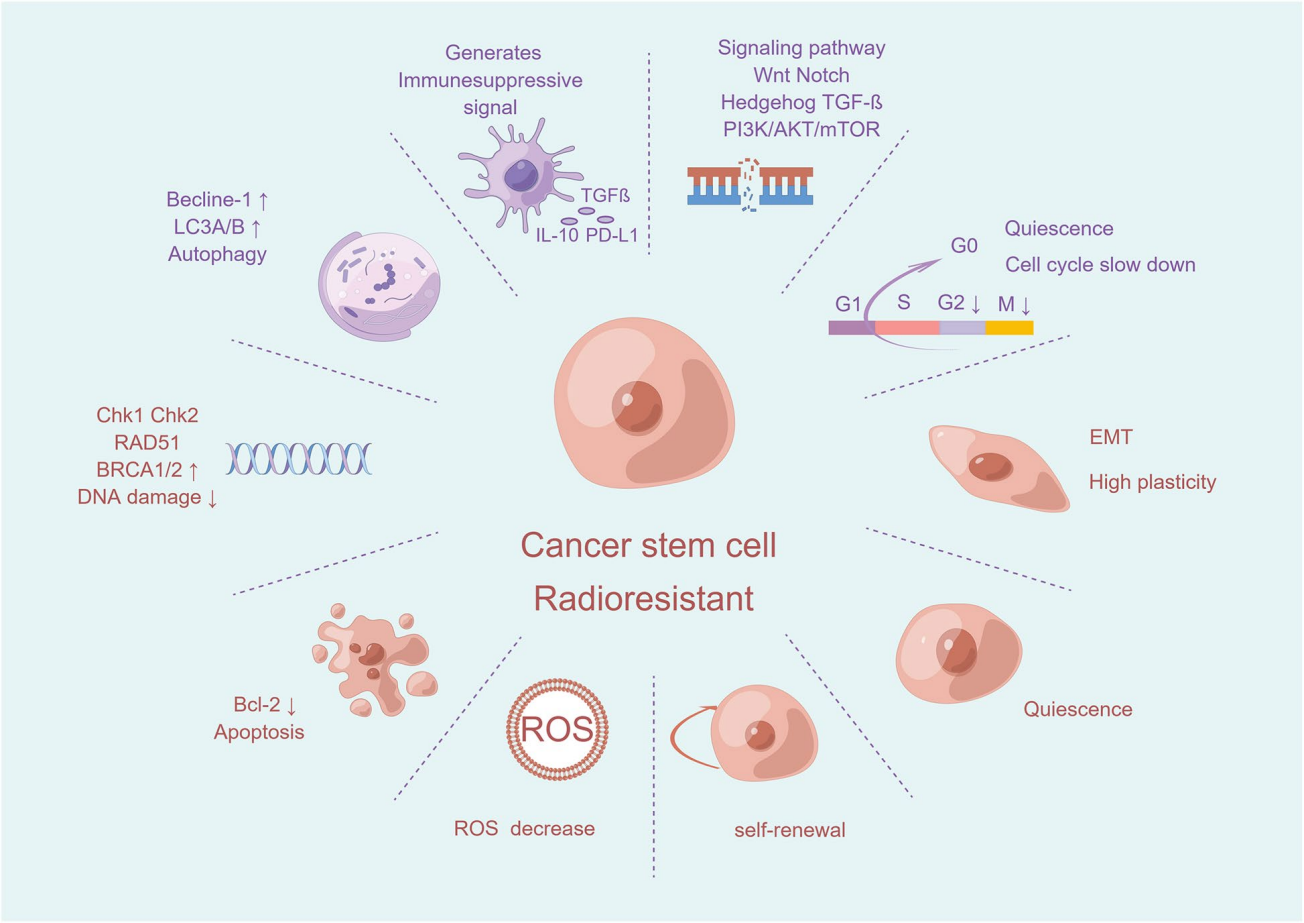
The mechanisms by which cancer stem cells generate radioresistance. This diagram shows how CSCs can self-renew upon differentiation, become quiescent, be involved in tumorigenesis, and generate immunosuppressive signals as well as exert possible effects of DNA damage repair, low ROS levels, apoptosis, autophagy, and epithelial–mesenchymal transitions in tumor stem cell–associated radioresistance. In addition, several active signaling pathways (e.g., Wnt Notch Hedgehog TGF-ß PI3K/AKT/mTOR) may also be closely related to tumor stem cell radioresistance. EMT: epithelial–mesenchymal transition, ROS: reactive oxygen species, TGF-β: transforming growth factor-β, PD-L1: programmed cell death ligand 1, IL-10: interleukin 10, Bcl-2: B-cell lymphoma-2

Tumor stem cells are a group of cells that continue to renew and differentiate themselves in tumorous tissues, thus producing a heterogeneous population of tumor cells [73]. Although they constitute a very minor proportion of tumor tissues, they are highly tumorigenic, and their capacity to regenerate themselves is unique. They also possess DNA repair, effective ROS scavenging, long-term dormancy, weak adhesion, and shape immune suppression properties in the tumor microenvironment throughout the tumor's development, which is a crucial factor causing tumor invasion and metastatic recurrence [183, 184]. Consequently, tumor stem cells are more radiotherapy-resistant than ordinary tumor cells [74, 75]. Increasing evidence supports the idea that cancer stem cells (CSCs) can resist the killing effects of radiation via different pathways [76–79, 185] (Fig. 3).

#### DNA damage repair and the cell cycle

After tumor tissue has been exposed to ionizing radiation, CSCs respond by activating checkpoint pathways, upregulating the expression of genes that mediate DNA repair and delaying cell cycle progression to allow time for repair, thereby evading the damaging effects of radiotherapy [186]. Multiple solid tumor CSCs reportedly have extensive DNA damage repair and delayed cell cycle progression because of the activation of the ATR-Chk1 and ATM-Chk2 signaling pathways [187–191]. By preferentially activating the DNA damage checkpoint response to control the cell cycle, boost DNA repair capacity, and decrease radiosensitivity by upregulating the expression of HR genes RAD51-, BRCA1/2-, and CD133-positive glioma stem cells help to develop glioma radioresistance [192, 193]. DNA repair capability as a measure of radioresistance may also be related to the effective ROS scavenging capacity of CSC’s, as they typically have low ROS levels [194, 195]. In addition, CSCs are usually quiescent in the cell cycle and are maintained in the most resistant G0 phase, which thus mitigates the DNA damage caused by radiation [81].

#### Autophagy

Autophagy is a lysosome-mediated process for the degradation of damaged and dysfunctional components and the recycling of metabolic substrates [82, 83]. Several studies on autophagy in tumor cells have shown that autophagy has dual therapeutic implications, with a possible oncogenic effect on normal stem cells in the early stages of tumor development, as well as a crucial role in maintaining the cancer stem cell phenotype, promoting tumor invasion, and protecting tumor cells from ionizing radiation damage during tumor development [84–86]. Autophagy protects hematopoietic stem cells from damage by harmful oncogenic substances such as ROS, however, once malignant transformation has occurred, autophagy provides protection to CSCs, counteracts radiotherapy toxicity, and helps leukemia stem cells survive [196]. The inhibition of the autophagy-related proteins SLC7A5/LAT1 and ATG5 reportedly increases radiosensitivity in HNSCC, suggesting that the induction of autophagy increases radioresistance in HNSCC [197]. Ionizing radiation can induce GSCs with a radiation-resistant stemness phenotype in GBMs by promoting autophagy via the Wnt/-catenin pathway [198]. Researchers discovered that lysosomal and autophagic levels were significantly increased in CSCs irradiated with FLASH-RT at ultra-high dose rates compared to those in normal cancer cell controls (MCF-7) and that, by activating autophagy, CSCs achieved a higher survival rate and were more resistant to radiation [80]. The targeted control of autophagy is thus a potential new technique to increase treatment resistance in CSCs; however, further research is required to better understand how autophagy interacts with CSCs.

#### Tumor microenvironment

CSCs reside in specific niches which protect them from radiation [199]. For example, solid tumor CSCs are often found in hypoxic niches [200], which can protect against radiation damage by reducing ROS production, reducing DNA damage, and activating the hypoxia-inducible factor (HIF) signaling pathway [201, 202]. In the hypoxia response system, HIFs are core regulators that open up survival-related signaling pathways such as TGF-β, Notch, Hedgehog, Wnt/β-catenin, and PI3K/AKT/mTOR [87, 203–206]. These facilitate the survival of CSCs in a hypoxic environment by maintaining the phenotype and properties of CSCs and by achieving self-renewal and invasive migration, which in turn leads to radioresistance [88–90, 159]. In addition, tumor stem cells can generate immunosuppressive signals via mutual signaling with the microenvironment, thereby shaping their microenvironment into an immunosuppressive state and generating a growth ecology conducive to tumor expansion, resulting in radioresistance [207]. Current research suggests that CSCs are closely related to tumor radioresistance and that a deeper knowledge of potential treatment targets could be an efficient strategy by which to decrease radiotherapy resistance.

### Metabolic reprogramming

**Fig. 4:**
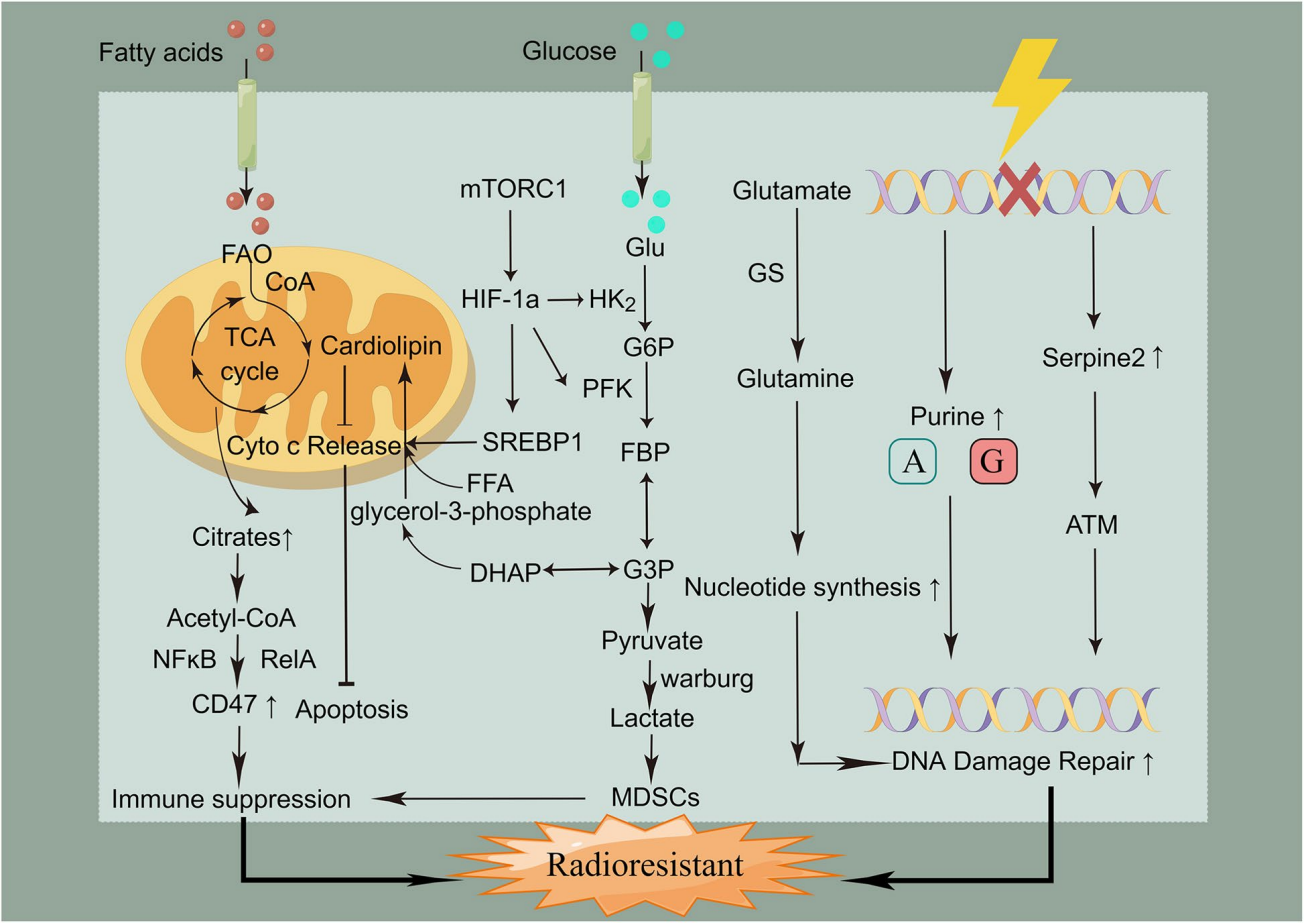
The relationship between metabolic reprogramming and radioresistance. Active glycolysis and lipid metabolism, which are typical of cancer metabolism, promote the development of radioresistance by mediating the development of immunosuppressive microenvironments and blocking apoptosis. In addition, the high expression of glutamine synthetase, purines, and serine protease inhibitor E2 can promote DNA damage repair, thereby leading to radioresistance. FAO: fatty acid oxidation, FFA: free fatty acid, Glu; glucose, G6P: glucose-6-phosphate, G3P: glyceraldehyde triphosphate, DHAP: dihydroxyacetone phosphate, FBP: fructose-1,6-bisphosphate, GS: glutamine synthetase, ATM: ataxia-telangiectasia mutated, NF-κB: nuclear factor-kappa B, Cyt c: cytochrome c, HK2: hexokinase 2, PFK1: phosphofructokinase 1, MDSCs: myeloid-derived suppressor cells, DHAP: dihydroxyacetone phosphate, HIF-1α: hypoxia-inducible factor 1α, SERPINE2: serine protease inhibitor E2, TCA cycle: tricarboxylic acid cycle

Cancer and metabolic diseases are intimately related [208, 209]. Metabolic reprogramming is a mechanism by which tumor cells rapidly adapt to hypoxia, acidity, and nutrient deficiencies to promote cell proliferation and is related to glucose, lipid, and amino acid metabolism and other metabolic pathways, which are themselves closely related to tumor development and radiotherapy resistance [210–213]. Glucose metabolic reprogramming is the most representative metabolic phenotype in tumors. Tumor cells have a unique energy metabolism that does not utilize their mitochondrial oxidative phosphorylation capacity even in the presence of oxygen, and this is a phenomenon of active aerobic glycolysis known as the Warburg effect, which is manifested by lactate accumulation and the simultaneous acquisition of ATP [214]. Studies have shown that radiotherapy can increase glycolysis in pancreatic cancer cells, leading to the secretion of high lactate levels that promote the functional activation of MDSCs in the microenvironment via the GPR81/mTOR/HIF-1α/STAT3 pathway. This further promotes a suppressive immune microenvironment, which in turn leads to tumor progression, recurrence, and radiation resistance [158]. In addition to the conventional glycolytic pathway that produces lactate and pyruvate, glucose can also stimulate cardiolipin synthesis in large amounts in hepatocellular carcinoma cells. This inhibits the release of cytochrome C in response to radiation stimulation and blocks the initiation of apoptosis, thereby contributing to the development of radiotherapy resistance [215]. Lipid metabolism reprogramming is a common aspect of cancer metabolism, with the combined aspects of enhanced lipogenesis, increased lipid content, and lipid-dependent catabolism coming together to support and guide tumor cell responses against radiotherapy [216, 217]. Metabolic rearrangements in tumor cells enhance mitochondrial fatty acid oxidation (FAO), which can provide cellular energy in the form of ATP via catabolism in mitochondria, helping tumor cells to escape radiotherapy-induced death and upregulate CD47 transcription via citric-acid–acetyl-coenzyme-A–RelA, which further exerts immunosuppressive effects and protects radiotherapy-resistant GBM cells from macrophage attacks. The combination of a FAO inhibitor and an anti-CD47 antibody also improved tumor treatments in a mouse model for GBM relapse after radiotherapy [218–220]. Furthermore, cell biology and animal studies have found that high expression of glutamine synthetase results in anti-apoptotic, pro-proliferative, and significantly increased resistance to radiation in tumor cells and that the knockdown of glutamine synthetase decreased the efficiency of the ab-initio synthesis pathway and slowed DNA damage repair [221]. In vivo and ex-vivo analysis of lung cancer revealed long durations of DNA damage marker γH2AX foci in serine protease inhibitor E2 (SERPINE2) knockdown cells, suggesting that SERPINE2 knockdown reduces DNA double-strand damage repair activity in lung cancer cells, thereby increasing radiosensitivity [222]. Wahl et al. found that increased purine levels in GBM promoted DNA repair and led to radiotherapy resistance, and that this was reversed with the inhibition of purine synthesis [223]. While experiencing radiotherapy damage, some tumor cells will have their metabolic characteristics altered by themselves or by the radiotherapy itself, leading to radiotherapy resistance. Therefore, understanding the relationship and regulatory mechanisms between metabolic reprogramming and radiotherapy responses is critical, as is selecting appropriate combination therapy strategies to improve radiotherapy efficacy (Fig. 4).

### Exosomes and non-coding RNA

**Fig. 5:**
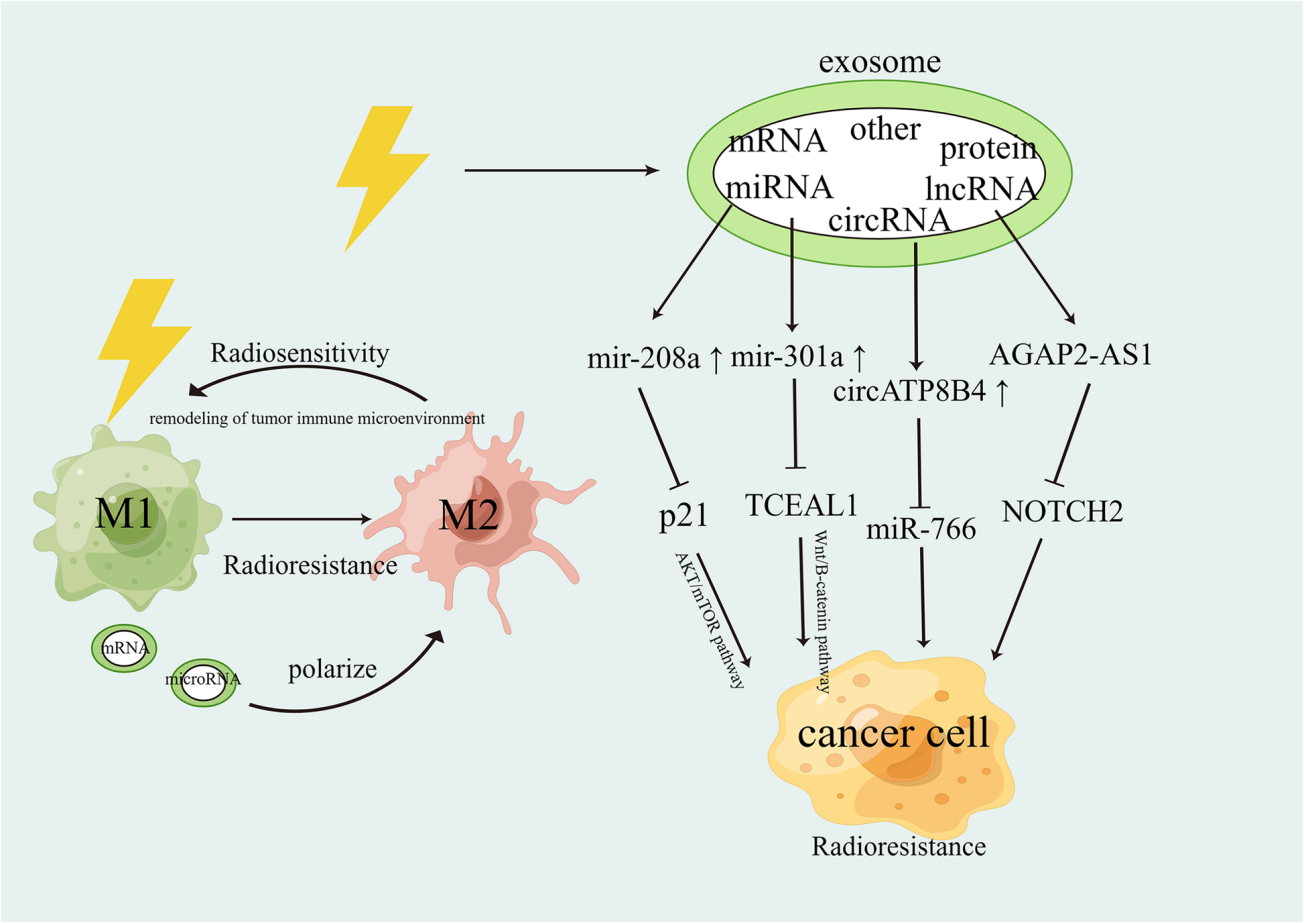
The role of exosomes in cancer radiotherapy resistance. Radiation-induced paracrine effects mediated by exosomes and their contents (e.g., exosomal proteins and non-coding RNAs) affect radiotherapy efficacy via different pathways. Radiation also promotes the polarization of M1 tumor-associated macrophages to the M2 phenotype, which suppresses the anti-tumor immune response, whereas M1 macrophage–derived exosomes repolarize the M2 phenotype to the M1 phenotype, reshaping the tumor immunosuppressive microenvironment and improving the efficacy of radiation therapy. M1: M1-type tumor-associated macrophages (anti-tumor), M2: M2-type tumor-associated macrophages (pro-tumor), miRNAs: micro-RNAs, lncRNAs: long non-coding RNAs, circRNA: circular RNA, mRNA: messenger RNA

Exosomes have recently received substantial attention because of their potential use in treating radiation damage. Exosomes are cellular vesicles ranging from 40–160 nm in size that contain a variety of compounds, including proteins, lipid metabolites, nucleic acids, and lipids [224, 225]. Exosomes, which are abundant in numerous bodily fluids and tissues, actively participate in both proximal and distant cell–cell communication, promote neo-vascularization, mediate the value-added invasion of tumor cells, and promote resistance to radiotherapy [226, 227]. Multiple lines of evidence suggest that exposure to RT increases the release of exosomes and can cause alterations in their contents, mediating bystander effects to secrete multiple factors that enhance radiotherapy resistance [228–233]. Non-coding RNAs—including miRNAs, lncRNAs, mRNAs, and circRNAs—are some of the most abundant components found in exosomes, and they are essential for radiation resistance and cancer progression [234, 235]. Researchers have found that the radiation-induced upregulation of exosomes containing miR-208A in lung cancer patients promotes cell proliferation by targeting p21 and the corresponding activation of the AKT/mTOR pathway in lung cancer cells, leading to radiation resistance [236]. According to Yue et al., GBM-secreted exo-miR-301a may be transmitted to comparable normoxic cultivated cells under hypoxic conditions. The suppression of TCEAL7 gene expression can be targeted with Wnt/-catenin signaling to improve radioresistance [237]. Another study on GBMs found that circATP8B4 was expressed at significantly higher levels in RR-EV (radioresistant U251 cells) than in Nor-EV. Furthermore, circATP8B4 from radioresistant exosomes of glioma cells could promote cellular radioresistance by acting as a microRNA (miR)-766 sponge when transferred to normal glioma U251 cells [238]. Zhang et al. demonstrated that the notch homologous protein 2 (NOTCH2), miR-296, and lncRNA AGAP2 antisense RNA 1 (AGAP2-AS1) axis can affect the development and radioresistance of lung cancer, and that the M2-macrophage-derived exosomal lncRNA AGAP2-AS1 reduces lung cancer radiosensitivity by lowering miR-296 and increasing NOTCH2 [239]. Endometrial cancer M2-macrophage-derived exosome hsa_circ_0001610 is overexpressed, which in turn mediates the upregulation of the expression of the cell cycle protein B1, thereby increasing the radioresistance of endometrial cancer cells [240]. Numerous studies have demonstrated that tumor-cell-derived exosomes can polarize tumor-associated macrophages (TAM) in an M2-like manner and accelerate the growth of tumors by secreting a variety of secretagogues [241–243]. However, recent investigations have discovered that exosomes produced by immune cells are crucial for radiosensitization. M1-type macrophage-derived macrophages can repolarize tumor-promoting M2-type TAMs to the anti-tumor M1 phenotype by secreting a variety of mRNAs and microRNAs, reshaping the tumor immunosuppressive microenvironment and improving radiosensitivity; engineered M1-type macrophage exosomes have shown outstanding therapeutic effects in subcutaneous transplanted tumors of mouse lung cancer [244]. The above studies suggest that exosomes and their contents influence cancer radioresistance via different pathways. Engineered exosomes have a wide range of applications in improving the efficacy of radiotherapy. In-depth future studies on exosomes will help to identify useful therapeutic targets and enhance the effectiveness of radiation **(**Fig. 5).

### Ferroptosis

**Fig. 6:**
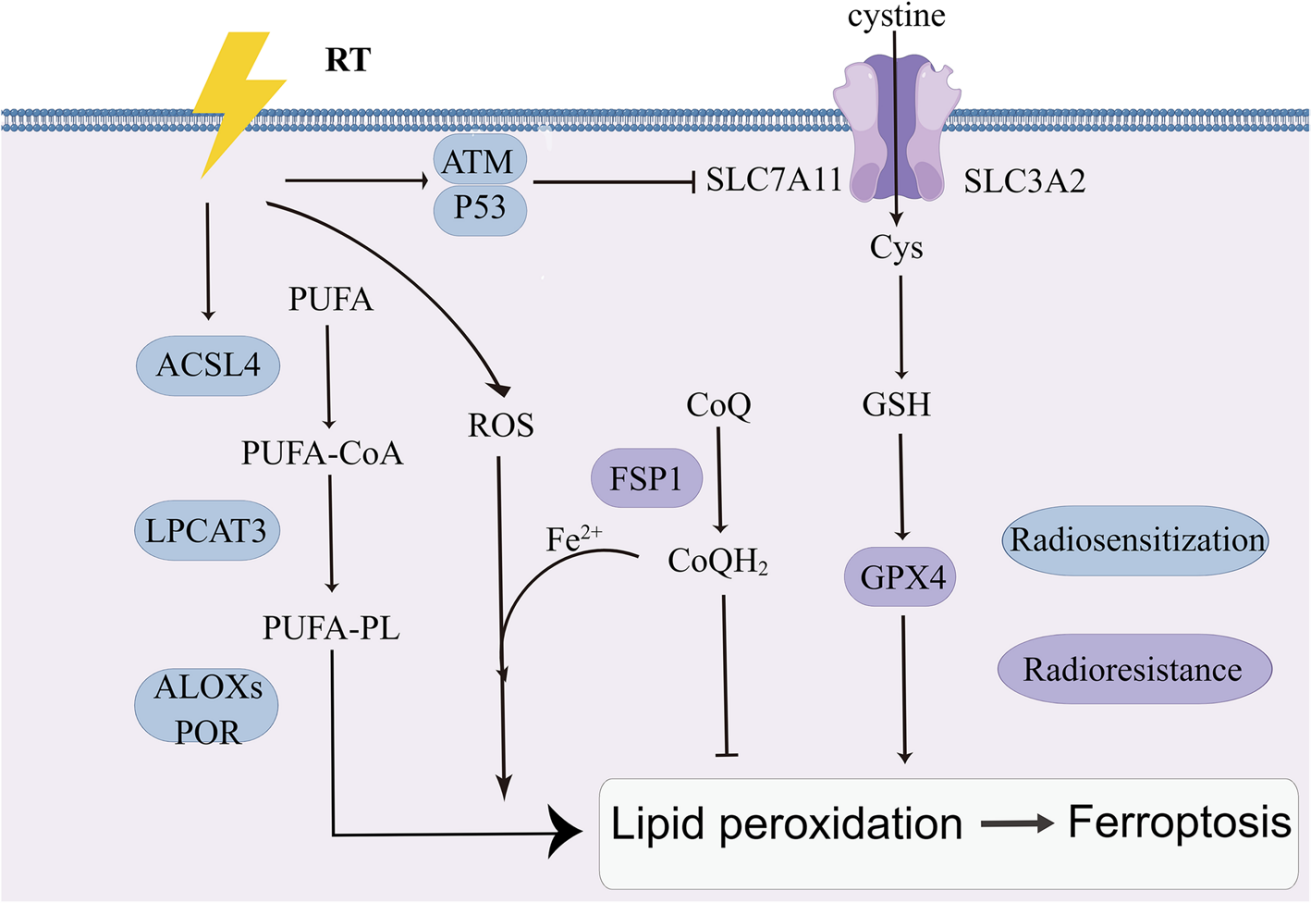
Pathways promoting iron-caused death, PUFA-PL synthesis, the GPX4 and FSP1-CoQ signaling axes, and iron metabolism. Iron-caused death facilitates increased radiosensitivity (high expression in the blue areas), and inhibition of iron-caused death helps cancer cells acquire radioresistance (high expression in the purple areas). ACSL4: acyl coenzyme A synthetase long chain family member 4, LPCAT3: lysophosphatidylcholine acyltransferase 3, POR: cytochrome P450 oxidoreductase, ATM: ataxia-telangiectasia mutated, FSP1: ferroptosis suppressor protein 1, Cys: cysteine, GSH: glutathione, GPX4: glutathione peroxidase 4, PUFA-PL: polyunsaturated fatty acid- containing phospholipid, ROS: reactive oxygen species, CoQ: ubiquinone, SLC7A11: solute carrier family 7 member 11, LPCAT3: lysophosphatidylcholine acyltransferase 3, SLC3A2: Solute Carrier Family 3 member 2, PUFA: polyunsaturated fatty acid, CoQH2: ubiquinol, ALOX: lipoxygenase

Ferroptosis differs physiologically and mechanistically from apoptosis, autophagy, and necrosis and is a new mode of cell death that is iron-ion-dependent and caused by the enormous accumulation of intracellular lipid ROS and tissues with redox balance [245]. Ferroptosis has recently generated a large amount of interest in the field of cancer research, and an increasing number of studies have revealed that ferroptosis plays a significant role in tumor therapy and the suppression of cancer [246–249]. Radiotherapy may induce regulated ferroptosis via multiple pathways, primarily iron metabolism, lipid metabolism, and the GPX4/System xc and GXP4 non-dependent pathways. Numerous analyses have shown that cancer cells can reduce the efficacy of cancer therapy by negatively regulating ferroptosis [250, 251]. miR-7-5p is highly expressed in clinically relevant radiotherapy-resistant cells and leads to radiation resistance by downregulating mitoferrin and reducing Fe2 + in the mitochondria, thereby inhibiting ferroptosis [252]. Lei et al. showed that radiotherapy can promote lipid peroxidation, and thus ferroptosis, by generating large amounts of ROS and upregulating the expression of ACSL4, and that the knockdown of ACSL4 in tumor cells leads to significant radioresistance. This group also found that radiation induced the overexpression of ferroptosis-inhibiting genes such as GLC7A11 and GPXA, and that the concatenation of ferroptosis-inducing agents in tumor models to inhibit GLC7A11 or GPXA produced a significant reversal of radiation resistance [253]. The protein p53, which is known as the “guardian of the genome,” is a common oncogene. Radiotherapy-mediated p53 activation promotes iron-caused death by reducing the expression level of the antioxidant system subunit SLC7A11, which inhibits glutathione synthesis; p53 deficiency causes radioresistance, in part via SLC7A11-mediated glutathione synthesis. When SLC7A11 is inhibited by ferroptosis-inducing agents (FINS), p53 mutant tumors become radiosensitive to it in vivo [254]. A recent study found that upregulation of FSP1 expression via NRF2 in KEAP1-deficient lung cancer cells leads to ferroptosis resistance and radiotherapy resistance, thus identifying an effective therapeutic strategy of targeting CoQ-FSP1 signaling to weaken ferroptosis defenses and overcome radiotherapy resistance caused by KEAP1 inactivation [255]. Ferroptosis resistance in cancer cells confers resistance to cancer therapy, thus disrupting the mechanisms that drive ferroptosis to produce resistance may re-sensitize tumors to radiation therapy. Further research into the relationship between radiotherapy and ferroptosis as well as the search for new relevant effectors could lead to the development of new oncology treatment approaches that can overcome the challenge of radiotherapy resistance (Fig. 6).

## Conclusions

One of the main obstacles in the treatment of locally progressed, recurring, and metastatic malignancies is radiation resistance. This article reviews the genetic basis of resistance to cancer radiotherapy and discusses potential approaches to improve cellular sensitivity by targeting relevant key genes, signaling pathways, and key features of cancer. Radiation can exert an “ionizing effect” on atoms and molecules, thus producing a series of oxygen radicals that can cause cell death or cancer. Aberrant DDR, cell cycle redistribution, a hypoxic tumor microenvironment, autophagy, metabolic reprogramming, ferroptosis, gene mutations, and dysregulated signaling pathways are the main mechanisms that induce radioresistance in cancer. As resistance to radiation is caused by multiple factors, it can occur via different regulatory pathways in different cell types; consequently, different molecular interventions or combination therapies are required to overcome it in different tumor subtypes. Further dissection of the molecular mechanisms underlying radiotherapy resistance and interactions with the tumor microenvironment could enhance cancer responses to radiotherapy. With an understanding of these fundamental issues, potential strategies to overcome radioresistance may be designed that could help to stage and stratify cancer patients to identify new, more effective, and specific radiosensitizer-radiation combinations and to provide new clinical treatment options.

## Data Availability

Not applicable.

## Abbreviations

RT: Radiation therapy
ATM: Ataxia-telangiectasia mutated
ATR: ATM and Rad3-related kinase
DDR: DNA damage response
DNA-PK: DNA-dependent protein kinase
DSBs: Double-strand breaks
NHEJ: Non-homologous end joining
GBM: Glioblastoma
GSCs: Glioma stem-like cells
CAD: Caspase-activated Dnase
NO: Nitric oxide
IAPs: Inhibitor of apoptosis proteins
HNSCC: Neck squamous cell carcinoma
CAF: Cancer-associated fibroblasts
ROS: Reactive oxygen species
HIF: Hypoxia-inducible factor
EMT: Epithelial–mesenchymal transition
MDSCs: Myeloid-derived suppressor immune cells
CSCs: Cancer stem cells
SERPINE2: Serine protease inhibitor E2
FAO: Fatty acid oxidation
TAM: Tumor-associated macrophages
FINS: Ferroptosis-inducing agents
